# An Uncommon Side Effect of Bupropion: A Case of Acute Generalized Exanthematous Pustulosis

**DOI:** 10.1155/2015/421765

**Published:** 2015-11-24

**Authors:** Hasan Tak, Cengiz Koçak, Gülben Sarıcı, Nazlı Dizen Namdar, Mehtap Kıdır

**Affiliations:** ^1^Department of Dermatology, Faculty of Medicine, Dumlupinar University, 43100 Kutahya, Turkey; ^2^Department of Pathology, Faculty of Medicine, Dumlupinar University, 43100 Kutahya, Turkey

## Abstract

Acute generalized exanthematous pustulosis (AGEP) is a rare inflammatory dermatosis characterized by multiple nonfollicular pustules that occur on erythematous skin. Despite its similarity to pustular psoriasis and association with fever and leukocytosis, AGEP typically heals quickly. Etiologically, drugs and viruses have been suspected in most cases. Here, we present a case of AGEP, in a woman, that developed 1 day after starting bupropion for smoking cessation, as a rare side effect of the treatment.

## 1. Introduction

Acute generalized exanthematous pustulosis (AGEP) is a rare inflammatory eruption characterized by the sudden development of multiple small sterile pustules on erythematous skin. It is accompanied by fever and leukocytosis. The disease has typical histopathological findings and recovers spontaneously within 15 days [1]. Mucosal membrane involvement occurs in about 20% of the cases and the majority of patients have mild oral lesions [2].

Etiologically, antibiotics, mostly aminopenicillins and macrolides, play a role in more than 90% of the cases [2].

The differential diagnosis of AGEP includes generalized pustular psoriasis, subcorneal pustular dermatosis, pemphigus foliaceus, toxic epidermal necrolysis, drug reaction with eosinophilia, systemic symptoms syndrome, and other follicular eruptions such as acneiform and bacterial folliculitis [1, 2].

Bupropion is a dopamine reuptake inhibitor that is used as an antidepressant and for smoking cessation. There are 28 reports of dermatological side effects from bupropion, including angioedema, erythema multiforme, Stevens-Johnson syndrome, exfoliative dermatitis, urticaria, and serum disease [3].

Here, we report a case of AGEP that developed as a rare side effect of bupropion.

## 2. Case Report

A 30-year-old woman visited our outpatient clinic with acute eruptions that appeared 4 days earlier on her face and trunk and then spread to her extremities. She took a bupropion tablet for smoking cessation 1 day before beginning of the eruptions and had a fever for the past 4 days. There was no history of psoriasis, previous drug allergy, or use of another drug with bupropion. She denied the use of any over-the-counter medications, supplements, or herbal remedies. She had not used a new soap, shampoo, or laundry soap before the skin reaction appeared.

**Figure 1:**
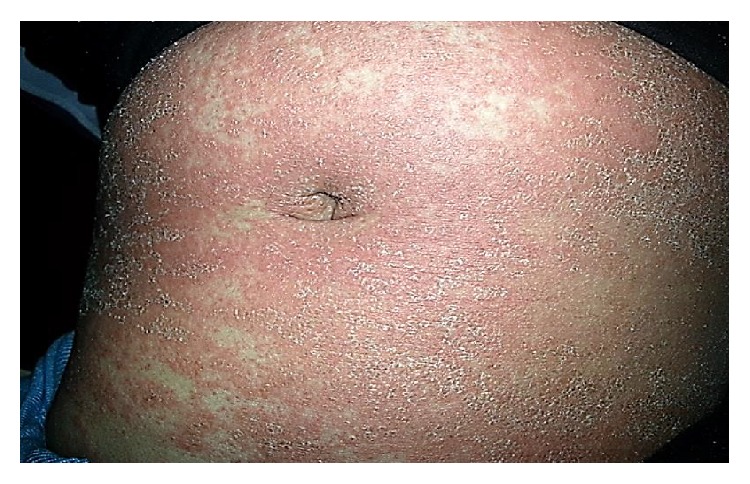
Revealed pustules on erythematous areas that tended to unite and did not display a follicular localization on the abdomen.

Dermatological examination revealed numerous pustules on her face, trunk, and legs. The erythematous areas tended to fuse and were not characterized by follicular localization (Figure 1). There were no lesions on the oral mucosa and the examination of other systems was unremarkable. Her axillary temperature was 38.2°C.

The laboratory results showed leukocytosis (12.90 × 109/L, 88.8% neutrophils) and an increased C-reactive protein level. There was no eosinophilia. Her liver enzymes, serum protein, albumin, and electrolytes were normal.

**Figure 2:**
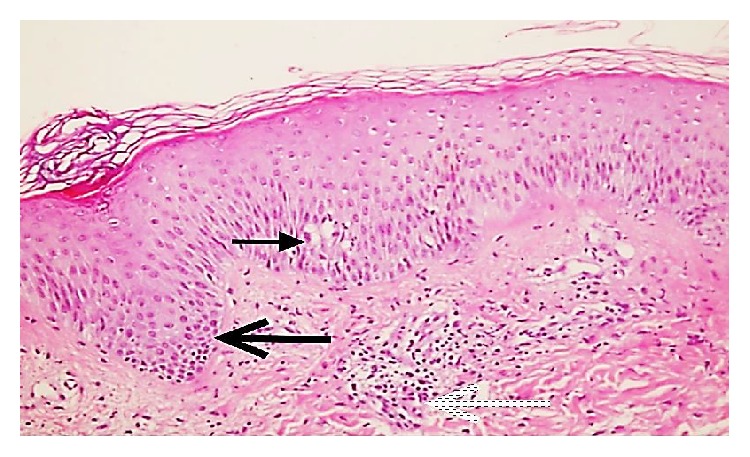
A photomicrograph of the biopsy showing neutrophilic pustular lesion together with epidermal spongiosis (thin arrow), minimal irregular acanthosis in epidermis (thick arrow), and neutrophilic and eosinophilic infiltration around dermal vessels (dashed arrow) (H&E ×200).

**Figure 3:**
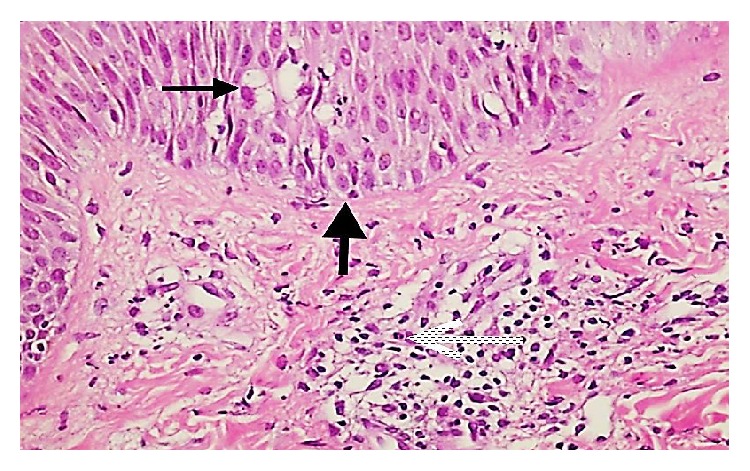
A photomicrograph of the biopsy showing neutrophilic pustular lesion together with epidermal spongiosis (thin arrow), minimal irregular acanthosis in epidermis (thick arrow), and neutrophilic and eosinophilic infiltration around dermal vessels (dashed arrow) (H&E ×400).

To confirm the diagnosis of AGEP and to rule out generalized pustular psoriasis, a 4 mm punch biopsy was taken from the skin. The histopathology showed neutrophilic pustular lesions (red arrow) together with epidermal spongiosis, minimal irregular acanthosis (black arrow) in the epidermis, and neutrophilic and eosinophilic infiltration (blue arrow) around dermal vessels (Figures 2 and 3).

The diagnosis of AGEP was made histopathologically, combined with the clinical findings (a fever for 4 days and an eruption that spreads from the face and trunk to the extremities) and a history of the absence of psoriasis or other drug use. The patient discontinued the bupropion treatment after the fever and eruption appeared. Intravenous methylprednisolone (40 mg/day) was administered for 4 days. In addition, topical corticosteroid and oral analgesic and antihistaminic were used. Within 4 days of the treatment, there were no new pustules and the healing was complete within 10 days with exfoliation.

## 3. Discussion

In 1980, Beylot et al. first described AGEP as a different entity from a drug eruption, characterized by sterile pustules on erythematous skin and usually confused with generalized pustular psoriasis [4]. Then, in 1991, Roujeau et al. outlined the characteristic features of AGEP in 63 cases [5]. These characteristic features were nonfollicular sterile pustules (5 mm) that were intraepidermal or subcorneal on histopathology (together with one or more additional findings like dermal edema, vasculitis, perivascular eosinophilia, or focal keratinocyte necrosis), disappearance of the eruptions within 15 days after drug cessation, presence of fever over 38°C, and neutrophilia over 7 × 109/L.

There are several theories on the pathogenesis of AGEP. Britschgi et al. suggested a T-cell mediated mechanism, as evidenced by positive findings on patch tests and lymphocyte transformation tests [6]. Moreau et al. proposed that AGEP is a delayed-type hypersensitivity reaction [7]. Another possible mechanism is the production of antigen-antibody complexes induced by an infection or drug that activates the complement system, which in turn leads to neutrophil chemotaxis [8].

In recent years, a new concept called pharmacological interaction has been developed to explain drug-induced hypersensitivity reactions. This concept implies direct, reversible interactions of the drug with T-cell receptors and is classified as a T-cell mediated reaction. Previous drug exposure is not necessary [9].

In our patient, since the reaction occurred within a single day of taking the first bupropion tablet, we believe that the pathogenesis of AGEP involves a pharmacological interaction or an unknown mechanism.

The EuroSCAR group has developed a validated scale for determining causation of AGEP by a medication [2]. This scale suggests that our case was definitely caused by bupropion, while the Naranjo algorithm [10] suggests that it was probably caused by bupropion. These results were similar to those of Ray and Wall [11].

Although viral infections [5, 12] or hypersensitivity to mercury [13] has been reported in the etiology, Sidoroff et al. suggested that drugs are more likely to trigger AGEP, and they found no relationship between infection and the development of AGEP [14].

A high proportion of AGEP cases have been attributed to aminopenicillins or macrolides but, interestingly, not to sulfonamides, which have a higher potential for causing other cutaneous drug reactions. Some cases have been attributed to antimycotic drugs. Moreover, several nonantibiotics, especially calcium channel blockers, carbamazepine, and paracetamol, have been reported as the culprit agents in numerous cases [2].

To the best of our knowledge, 129 different drugs have been implicated in the etiology of AGEP [3]. Recently, tigecycline and labetalol and psychotropic drugs such as amoxapine, sertraline, and bupropion were added to this list [3, 15, 16]. When we searched the literature for an association between bupropion and AGEP, ours was the second reported case [11].

The differential diagnosis of AGEP includes generalized pustular psoriasis. Although the pustules in the two diseases cannot be distinguished clinically, histopathological examination shows widespread edema in the dermis, vasculitis, perivascular eosinophilic infiltration, and focal keratinocyte necrosis in AGEP, while the presence of regular acanthosis in the epidermis supports pustular psoriasis [17, 18]. In our case, the minimal irregular acanthosis, widespread dermal edema, and perivascular lymphocytic and eosinophilic infiltration were thought to favor a diagnosis of AGEP.

The treatment of AGEP involves stopping the causative drug and supportive treatment for the symptoms and local lesions. Systemic corticosteroids are not required in most cases [2].

Acute generalized exanthematous pustulosis should be included in the differential diagnosis of a patient with a sudden-onset widespread pustular eruption. In such patients, a history of psoriasis and drug use should also be investigated. We also need to consider bupropion as the cause of these cutaneous side effects.
